# Expert Perspectives on the Performance of Explosive Detection Canines: Operational Requirements

**DOI:** 10.3390/ani11071976

**Published:** 2021-07-01

**Authors:** Brian D. Farr, Cynthia M. Otto, Julia E. Szymczak

**Affiliations:** 1 Army Medical Department Student Detachment, 187th Medical Battalion, 32nd Medical Brigade, Joint Base San Antonio-Fort Sam Houston, San Antonio, TX 78234, USA; 2 Penn Vet Working Dog Center, Clinical Sciences and Advanced Medicine, School of Veterinary Medicine, University of Pennsylvania, Philadelphia, PA 19104, USA; cmotto@vet.upenn.edu; 3 Department of Biostatistics, Epidemiology and Informatics, Perelman School of Medicine, University of Pennsylvania, Philadelphia, PA 19104, USA; jszymcza@pennmedicine.upenn.edu

**Keywords:** explosive, detection, canine, performance, requirements, deployment

## Abstract

**Simple Summary:**

Explosive detection canines safeguard lives and property by searching out and identifying explosive threats. The handlers, trainers, and leaders who work closely with these canines best understand what they are asked to do when tasked to search for explosives. This study used interviews with these highly experienced individuals to identify the requirements of explosive detection canines in team performance and in the physical, climate, operational, and explosive odor environments. These canines are used in different ways, and these differences influence their requirements. Despite these utilization differences, many requirements common to all explosive detection canines were identified. Searching for explosives is a team effort, and the handler likely has the greatest influence on the canine’s performance. A key requirement is appropriate preparation for the expected operational environment. The results of this study can inform the training, assessment, and utilization of these canines and guide future research in explosive detection canine research.

**Abstract:**

Explosive detection canines (EDC) play an important role in protecting people and property. The utilization of and research on EDCs is often based on personal experience or incomplete knowledge. EDC practitioners (handlers, trainers, and leaders) possess the institutional knowledge necessary to understand EDC operational requirements. This study utilized a qualitative approach with semi-structured interviews of EDC experts (*n* = 17) from across the employment spectrum. The interviews elicited EDC expert perceptions of the performance of the EDC team and the operational requirements in the physical, climate, operational, and explosive odor environments. Analysis of the data revealed commonalities across all EDCs and utilization-specific differences. To be effective, the EDC team must function well on both ends of the leash, and the handler likely has the greatest impact on the EDC’s performance. Common requirements include expectations to perform at a high level in a variety of manmade and natural physical environments and under a range of climate conditions. EDCs must work through the visual, olfactory, and auditory challenges of the operational environment and the countermeasure efforts of those utilizing explosive devices. Utilization-specific differences like patrol or assault training and utilization add additional requirements for some EDCs. The results of this study can be used to inform EDC selection, training, assessment, and deployment, and further research into EDC performance.

## 1. Introduction

Explosive detection canines (EDCs) play a critical role in protecting people and infrastructure from explosive threats [1,2,3,4,5]. EDCs differ from other detection canines, as the work of searching for explosives is repetitive, rarely rewarding, and associated with lethal consequences [6,7]. The EDC team is a mobile sensor solution consisting of paired mammals, with the handler and EDC both playing important roles. The end goal is a consistent level of performance in the face of the challenges present in the operational environment. Effective selection, training, assessment, and utilization are key steps towards that goal, and these processes must be conducted in an operationally realistic manner [8,9,10].

To date, no one to our knowledge has published a systematic study examining EDC operational requirements. Thus, selection, training, assessment, and utilization of EDCs is often based on personal experience or incomplete descriptions of these requirements. Much of the institutional knowledge associated with EDC operations exists as tacit knowledge that is shared and analyzed informally by EDC practitioners: handlers, trainers, and leadership [11,12,13]. This knowledge is often overlooked and difficult to articulate and transfer to other EDC practitioners and to EDC researchers [14,15,16,17,18,19]. Despite these challenges, the hard-earned perspectives of these individuals should be added to the growing body of EDC knowledge.

The goal of this study was to describe the operational requirements of EDCs. Subject matter expert interviews with EDC practitioners were used to identify explicit knowledge and elicit tacit knowledge associated with EDC operations [20,21,22]. Highly experienced individuals from a range of employment and experience backgrounds were included.

## 2. Materials and Methods

### 2.1. Study Design and Participants

In-depth, semi-structured interviews with EDC experts from four utilization sectors (law enforcement, military, federal, and private) were conducted. These sectors were selected as they represent the primary domains in which EDCs operate. This qualitative study was led by a working dog veterinary practitioner (BDF) with expertise in EDC care, training, and program management and graduate training in qualitative research, in collaboration with a working dog veterinary practitioner (CMO) with expertise in detection dog research, and a medical sociologist (JES) with expertise in mixed-methods research. We sought to create a purposive sample that included individuals in each EDC utilization sector with a broad range of experience as a handler, trainer, and/or leader. Subject matter experts were identified through key individuals at representative organizations that employ, train, or manage EDC teams for each utilization sector. These experts were recruited by email, and no incentive was offered for participation. The protocol was deemed exempt by the University of Pennsylvania Institutional Review Board.

### 2.2. Data Collection

Semi-structured interviews were conducted from April to May 2020. The interview guide contained open-ended questions intended to elicit participant knowledge and perceptions of operational requirements of EDCs (see Supplementary Materials for the guide). The key domains in the guide were the participant’s experience with EDC team performance and the physical, climate, operational, and explosive odor environments. One interview was conducted in person, and the remainder were conducted via telephone by BDF. Prior to the start of the interview, participants were made aware of the study’s purpose to characterize the operational performance requirements of EDCs. The interviewer assured potential participants that any sensitive or classified information shared during the interview would be redacted from the final dataset and no personally identifiable information would be collected. With permission of the participant, the interview was audio recorded.

### 2.3. Data Analysis

All audio files were transcribed and uploaded into NVivo 12 software for coding [23]. Data were analyzed using a flexible coding approach by one coder (BDF) in conjunction with two collaborators [24]. The interview guide was reviewed to guide the development of an index codebook, which was applied by BDF to two transcripts. The study team reviewed the application of the codebook to the data and suggested revisions. Discrepancies were resolved by consensus. Once the final codebook was developed, it was applied, line by line, to all transcripts. Once all transcripts were coded, patterns of themes within key domains were examined across interviews. Repeated themes across respondents were summarized and grouped them into two distinct categories: operational requirements of EDCs and performance degrading factors. The results reported in this manuscript are themes that were endorsed by the majority of participants.

### 2.4. Security Review and Omitted Data

The analyzed data were reviewed by the appropriate Department of Defense security offices (Joint Base San Antonio-Fort Sam Houston, San Antonio, TX, USA) and by individuals experienced in EDC operations. Any data determined to be sensitive or classified were omitted.

## 3. Results

### 3.1. Characteristics of Study Participants

Interviews were conducted with 17 EDC experts. Over half (*n* = 11, 64.7%) of the participants had experience as an EDC handler, and nearly all had experience as an EDC trainer (*n* = 14, 82.4%) or in leadership over EDC teams (*n* = 14, 82.4%). The majority of participants had experienced employment in multiple occupational settings, with 52.9% (*n* = 9) having experience in the military, 29.4% (*n* = 5) in law enforcement, 23.5% (*n* = 4) in the federal sector and 70.6% (*n* = 12) in the private sector. Participants with experience as an EDC handler, trainer, or leader had an average of 8.0 (range 1–17), 18.2 (range 2–35), and 17.6 (range 2–25) years of experience respectively. Participants with an experience as an EDC handler or trainer had experience in these roles an average of 14.3 (range 0–28) and 5.4 (range 0–23) years ago, respectively, and all participants with experience as a leader were currently in that role. Many of the participants had additional experience selecting and procuring potential EDCs, certifying, and auditing EDCs and EDC teams, consulting, teaching, participating in EDC working groups, and conducting EDC-related research, development, testing, and evaluation. Interviews ranged in length from 36 to 74 min, with a median of 58 min. In the sections that follow, exemplar quotes to support key themes will be provided in the accompanying tables.

### 3.2. EDC Utilization

Participants reported that EDCs are utilized in many ways, some of which are unique to one employment type, and others that are common to multiple employment types. An EDC may be utilized in just one way or in multiple ways during its career. A single EDC may be asked to perform multiple roles during a single shift or operation. Some EDCs also perform criminal apprehension or assault roles, and these roles may occur immediately before, during, or immediately after searching for explosives.

EDCs were described by participants as generally being used in two roles: screening objects or clearing areas for explosive threats. Screening typically consists of searching numerous discrete objects to confirm the absence of explosives. An EDC screening for explosives may be mobile, stationary, or a combination of the two. Screening operations may be continuous or intermittent. If screening operations are continuous, multiple EDCs may be assigned in rotation with a fixed or flexible work-to-rest ratio.

Clearance usually involves moving through a building, area, or large vehicle to identify explosives or confirm the absence of explosives. Participants explained that clearance operations may be categorized by the probability of identifying an explosive. For law enforcement EDCs, higher probability clearance operations include responding to bomb threats or searching suspicious packages. Military, law enforcement, or federal EDCs may perform clearance of routes or buildings with a pre-identified risk of explosives or explosive devices (Table 1, Quote 1). Lower probability operations include clearing a building, residence, or vehicle(s) for a dignitary or special event (Table 1, Quote 2).

### 3.3. EDC Team Performance Requirements

In the EDC team, participants stated that both the EDC and the handler must demonstrate high levels of performance to be operationally successful (Table 2, Quote 3). EDCs must be healthy, highly motivated to search, and utilize appropriate search behaviors. An EDC must have its basic needs satisfied (e.g., hunger, thirst, and need to eliminate) and be free of performance-limiting medical conditions (e.g., illness, lameness, or pain). Participants stated that EDCs must possess sufficient athleticism to navigate the search area and endurance to effectively search for the required duration (Table 2, Quote 4). Hunt drive was described by participants as the primary drive necessary for detection. They stated that hunt drive is characterized as motivation, a consistent high level of energy, focus on the task, and ignoring or rapidly recovering from distractions (Table 2, Quote 5). The performance of EDCs with appropriate hunt drive, it was stated, does not decrease during prolonged searches or repeated searches of the same or similar areas without identifying the target odor (Table 2, Quote 6). The reported appropriate search behaviors include a high rate of closed-mouth sniffing (Table 2, Quote 7), a methodical search of productive areas (e.g., door seams) (Table 2, Quote 8), and searching at a pace appropriate for the expected scent picture (e.g., slower when searching small packages) (Table 2, Quote 9).

The interaction between the EDC and their handler was identified by participants as a key component of operational performance. The EDC must have a good rapport with their handler and take direction easily. However, they must also confidently work independently of the handler, a skill that allows for effective searching at a distance (Table 2, Quote 10). Participants suggested that handlers must be conscious of their EDC’s physical, mental, and emotional needs, stay alert to changes in effective searching, and provide motivational training as needed if operationally feasible (Table 2, Quote 11).

### 3.4. Physical Environment Requirements

Participants explained that EDCs are expected to perform in any physical environment that does not pose an unreasonable risk of physical harm, such as the presence of hazardous chemicals, raw sewage, or fall risk from elevated surfaces (Table 3, Quote 12). Given that explosive devices can be placed, stored, or transported essentially anywhere, EDCs must deploy into a range of physical environments. In describing these environments, we found that participants’ answers could be categorized into manmade settings and natural settings. Manmade settings could be subdivided into indoor, outdoor and vehicles:

- Indoor–residential, industrial, storage, educational, commercial, entertainment venues, sporting venues, places of worship, and transportation terminals;
- Outdoor–exterior of indoor environments, damaged manmade structures, culverts and ditches, vehicle paths and tunnels, pedestrian paths and tunnels, transportation areas, and access control points;
- Vehicles.
  1. Small passenger–motorcycles, sedans, pickup trucks, and vans;
  2. Commercial–semi-trucks with flatbed or enclosed trailers, maritime vessels, and cargo planes;
  3. Mass transit–buses, trains, passenger planes, and ferries.

Participants described features of the natural physical environment in which EDCs are expected to search. These environments include those near manmade features and those far away from them, depressed areas, elevated and high-altitude areas, and the angled sides of terrain features (Table 3, Quote 13). Outdoor areas range from the yards of residential homes to parks and vast open landscapes with variable amounts and types of vegetation present, including rock, dirt, grass (short and tall), cactus-filled, dense forest, or desert. EDCs are expected to search along natural and manmade bodies of water (e.g., ponds, streams, rivers, and beaches) and to identify explosives under water (landmines under up to 18 inches of water and explosive caches under up to 3–4 feet of water). Natural vehicle and pedestrian paths (e.g., dirt paths or cross-country) are also locations for detection.

### 3.5. Climate Environment Requirements

Similar to their responses to the physical environment requirements, participants stated that EDCs must perform in nearly any climate based on their assignment or anticipated deployment as long as the climate is determined to be safe for the handler and other personnel to operate in. Situations with extreme heat, extreme cold, and with nearby lightning were identified as exceptions (Table 4, Quote 14). Some organizations strictly operate locally, so their EDCs would be expected to perform in a narrower range of climates. Other organizations operate predominantly indoors (e.g., at airports), but their EDCs are still expected to perform in the prevailing outdoor climate when necessary. Many EDCs are expected to travel great distances and operate in climates starkly differently from their home climate. Other EDCs encounter and are expected to perform in niche environments, including poor air quality and extreme heat or cold.

Participants explained that the EDC, handler, and leadership all play important roles in supporting operational performance in the climate environment. The EDC must be physically fit enough to thermoregulate and navigate the physical environment while simultaneously detecting effectively. The EDC must be properly acclimated to the climate environment, especially in hot and/or humid conditions. The EDC must be physically hardy enough and behaviorally acclimated to handle environmental changes (e.g., rain or snow) without an alteration in performance. The handler must be comfortable in all expected climates to maintain a consistent demeanor with the EDC and must ensure the EDC maintains the appropriate level of physical fitness and is properly acclimated to the climate. The handler must be able to monitor the climate and their EDC’s performance, implement corrective actions (e.g., rest), and make recommendations to their leadership regarding the EDC’s current and predicted performance. The role of EDC leadership is to establish climate-specific protocols to guide EDC utilization, effectively plan for the prevailing climate, and ensure adequate time for acclimation prior to utilization (Table 4, Quote 15).

### 3.6. Operational Environment Requirements

In addition to being able to perform in a diverse array of environments, participants explained that EDCs must display certain characteristics to ensure consistently high levels of performance in variable, volatile, and hyperstimulating environments. EDCs must be comfortable around all anticipated components of the environment, ignore distracting stimuli, and behave with innate confidence. When startled or distracted by a novel or noxious stimulus, an EDC must rapidly settle and effectively refocus on the detection task (Table 5, Quote 16). EDCs must operate consistently and without interference around people, animals, objects, sounds, smells, modes of transportation, in the context of time pressure, and requirements for situation-dependent responses:

- People (types)—bystanders, criminal suspects or detainees, familiar or unfamiliar supporting personnel, and healthy, injured, or deceased people;
- People (responses to EDC)—neutral, avoidant, anxious, and confrontational;
- Animals—domestic, feral, and wild;
- Sound—novel, sudden, sustained, loud, and high-pitched;
- Novel objects—rolling baggage, mobility devices (e.g., wheelchairs or walkers), and heavy machinery (e.g., forklifts);
- Odors—noxious (e.g., diesel fuel), distracting (e.g., food and animal urine), and similar to explosives (e.g., fertilizer).

EDCs may have limited time to acclimate to any of these stimuli if they are encountered for the first time operationally. Participants stated that the way the search is started, proceeds, and ends need to be considered when determining operational requirements. The EDC team may encounter repetitive and potentially monotonous searches or be expected to perform under significant time constraints due to altered plans or tactical pressure. The EDC may be transported to the search location via ground vehicle, aircraft, or watercraft. They may be lowered from a helicopter, attached to their handler as they fast rope or rappel, or they may travel on foot. Finally, the EDC may be expected to provide subtle final trained responses (e.g., behaviors indicating completion of the search). In some circumstances, EDCs may not be allowed to work to the source of the odor before the search is terminated due to tactical limitations.

Participants described handler characteristics that are required as EDCs navigate these environmental features. Handlers must be comfortable in the operational environment to continue monitoring the performance of their EDC and directing them as needed, while balancing any concurrent tactical situation and potential threat to their safety or that of the EDC (Table 5, Quote 17). The handler must also have the situational awareness to remain alert for visual indicators of explosive threats. Participants believed that EDC leadership plays a role in facilitating optimal performance through the provision of adequate time and resources to prepare the EDC team prior to utilization (Table 5, Quote 18).

EDCs that are trained to also perform criminal apprehension or assault functions face additional requirements. These EDCs must effectively detect explosives while cues around them indicate the opportunity to search for and engage a human is imminent (Table 5, Quote 19). They must also rapidly transition from engaging with a human back to searching for explosives (Table 5, Quote 20).

### 3.7. Explosive Odor Environment Requirements

Participants stated that EDCs must be able to detect all expected explosive odors in the anticipated operational presentations. They must be able to generalize sufficiently to detect odors similar but not identical to what they were trained with (e.g., homemade explosives with alternative ingredients or conventional explosives with regional differences). In addition, they must be able to discriminate between background odors and potential explosive odors (e.g., petroleum fuel from ammonium nitrate fuel oil) to prevent false positive responses (Table 6, Quote 21). An EDC must have a low enough detection threshold to identify explosive odor at the levels associated with operational threat devices. The handler must be experienced with and alert to subtle changes in behavior associated with scent pictures near their EDC’s threshold.

Explosives may be buried, emplaced high above the ground, located deep within a wall, or located in or on a stationary or moving person or vehicle (Table 6, Quote 22). The EDC, therefore, must be accustomed to searching for explosives in varied locations. The handler must be able to adjust the search strategy and direct their EDC appropriately.

## 4. Discussion

The purpose of this study was to identify the operational requirements of EDCs. This study utilized a qualitative approach to systematically identify the institutional knowledge accumulated by EDC subject matter experts [25]. Semi-structured interviews with open-ended questions and several participants from each EDC utilization sector revealed both common and unique operational requirements.

The qualitative approach is both effective and necessary to document the explicit and illuminate the tacit knowledge known primarily by EDC handlers, trainers, and leadership [25,26,27]. Individuals who possess this expert knowledge have not systematically shared detailed insights in such a comprehensive manner prior to this study, contributing to scientific understandings of EDC operational requirements. Future EDC research (and research into working dog performance in general) can leverage these findings, and researchers should be cautioned against relying on personal or limited experience when dealing with EDC operational requirements [28,29]. The participants were in near-unanimous agreement on EDC operational requirements and revealed the impact of utilization type on operational requirements. With few exceptions, EDCs have similar requirements in the physical, climate, and operational environments. The primary differences were in degree rather than in kind. For example, all EDCs are expected to recover quickly after exposure to sudden and loud sounds, but only some must continue to detect effectively after exposure to gunfire [30,31]. The way each EDC is used greatly affects the requirements it must fulfill. EDCs screening baggage and people at the airport do not face the same environmental and tactical pressures as military EDCs clearing routes and buildings during raids. Conversely, the military EDC supporting tactical operations typically experiences far fewer missions than the airport EDC performing continuous detection for many hours each day. Any use of operational requirements (e.g., to guide assessment or research) must consider both the general and utilization-specific requirements [32].

While the EDC team is a currently irreplaceable capability, the components on both ends of the leash must perform to the same high level to be effective. Many of the interview questions were aimed at the performance of the EDC, but participants consistently brought up requirements specific to the handler. Effective detection is a team effort, operational requirements must include the handler, and the handler likely has the greatest positive or negative effect on the EDC’s performance [33,34,35,36,37].

Participants emphasized the requirement for preparation in each environment. Nearly everything an EDC could encounter during a search is known ahead of time, allowing for and mandating effective preparation [38]. Certain factors (e.g., the presence of animals, specific sounds, and human behavior) cannot be anticipated, but the handler must be prepared to continuously monitor the performance of their EDC and apply corrective action when necessary.

Some of the themes that emerged from this study agree with the existing literature (e.g., the responsibility of the handler) [33,34,35,36,37]. Other themes (e.g., the supremacy of hunt drive) contrast with or are more narrowly focused than indicated by previous research [8,9]. A few areas (e.g., detection in the presence of cues for apprehension or assault or after performing these functions) have not been explored in the literature. The overlap between expert opinion and quantitative research highlights the importance of these areas and illustrates where aspects of the literature are used operationally. The gap between the experience of practitioners and the literature reveals the need for either additional research or greater communication of existing research to the operational level. Finally, the themes revealed in this study that have not been empirically studied represent areas for future research.

An additional consideration is that public dissemination of the operational requirements of EDCs risks revealing critical information that may be of value to adversaries [39,40]. This study utilized multiple screening steps to minimize this risk while attempting to maximize the value to the scientific literature and future research. Future research into EDC capabilities and limitations should consider a similar approach.

The strengths of this study were the expert interview approach and the inclusion of highly experienced participants from diverse backgrounds. These factors resulted in documentation of institutional knowledge previously absent from the literature. Use of a qualitative approach limits the ability to generalize these findings to all EDC operations. However, the diverse experiences of the participants, the high degree of agreement in their answers and the detail with which they shared their knowledge has generated rich and detailed insights that can inform future research. The study only included U.S.-based participants, but these findings provide a starting point for future regional and comparative efforts.

Within the context of these limitations, these results can inform further study of the operational requirements of EDCs. This future work can focus on specific organizations (e.g., the military) or EDC utilization types (e.g., cargo screening). EDC leadership may benefit from leveraging these results to develop operationally realistic assessments and performance standards. Finally, these results could be used to develop a quantitative instrument for use with current EDC handlers [41] and then triangulated with existing EDC regulations and training material [26,42,43].

## 5. Conclusions

EDCs perform key roles in support of peace, and the knowledge critical to understanding EDC operations is predominantly held by EDC handlers, trainers, and leaders. This study illuminated the perspectives of these expert EDC practitioners in the pursuit of defining EDC operational requirements. While these requirements differed by utilization type, the study participants revealed commonalities that transcended utilization type. EDCs are expected to perform at a high level in a variety of manmade and natural physical environments and under a range of climate conditions. They must work through the visual, olfactory, and auditory challenges of the operational environment and the best efforts of an intelligent adversary. These results can be used to guide selection, training, assessment, and utilization, and to inform further research into EDC performance.

## Figures and Tables

**Table 1:** EDC utilization interview themes and exemplar quotations.

Interview Themes	Exemplar Quotations
High probability clearance	Quote 1. Any time that they’re doing a building entry for like a SWAT (Special Weapons and Tactics) team, a lot of times we’ll have the dogs pre-deployed to make sure. Here we had [adverse event prior to building entry]. So now beforehand, if a department has a dog, they’ll deploy the dog to the outside of that building to clear for the threat of any kind of explosive devices before the SWAT team goes into the breach. That’s a very common one here.
Low probability clearance	Quote 2. So, the difference in this one is that, if we’re going to do a facility sweep for dignitaries, then we evacuate the facility of personnel, so it’s a sensory free environment I should say, when it comes to mobile human stimulus or conversation stimulus. So, it’s usually quiet. It’s usually just the handler and the dog, and there can be somewhat of a playful environment in there. The dog just maneuvers through what is an otherwise dead space.

**Table 2:** EDC team performance interview themes and exemplar quotations.

Interview Themes	Exemplar Quotations
EDC team composition	Quote 3. Well, you never have a thing as just a dog. So, there’s a problem with this. It took us years to get non-dog people to think about, “Well I got my dog, the dogs are over here, we’re good.” It’s like, “No, you don’t have anything.” If you have a dog and you don’t have a qualified team, you don’t have anything. You have something that’s eating dog food. So, every dog is different, every handler is different. You have to ensure that the handler is properly qualified. He really knows what he’s doing with the dog. And then you’ve got to put the dog and the handler together and run assessments against that team. Once you say, “Okay, this team is qualified”, then you can say you have something. But until you get to that step, you don’t have anything. Or you have something, but you don’t know what you have.
Physical fitness and energy	Quote 4. Now, I’m not asking for a spaz, but I want to see some energy and some physical ability. Yeah, so the perception of physical fitness, I guess. If I see these dogs that are just kind of coming in and just very lazily approaching the search, although I’ve seen them be successful from time to time, that doesn’t instill a lot of confidence in me if I were forming an opinion about a dog or a team. I guess that’s energy slash drive slash physical conditioning.
Effective searching	Quote 5. Well, I think you’re seeing the drive of the dog. Is the dog actively searching? Is the dog searching in a systematic pattern? Is the dog searching productively? Unfortunately, it’s dictated often by the environment, but is the dog panting so you know that it’s not actively searching? Is the dog distracted by other things within the environment? I think all of those things would be important.
	Quote 6. I want to see a dog that can work an entire problem and not find anything, and then come back in and still be searching the same way. There’s no drop in their ability to want to go to work, do another search.
	Quote 7. Probably the one that stands out the most is active sniffing behavior. I’ve seen a lot of dogs over the years and all that, there’s nuances and differences between them, but the basic is, that the dog has to have active sniffing behavior. Sometimes just by watching the dog, you can see whether or not the dog has drive for the task.
	Quote 8. So, if it’s a desk, an office desk, if you will, there’s a lot of drawers, cabinets, whatever the case may be, the dog has to make sure that they’re sniffing all those drawer seams or cabinet door seams and all that to make sure that that’s where the odor’s going to be coming out of. The handler has to be observant in making sure the dog is hitting those productive areas. If he doesn’t, that’s where the handler has to come in and actually present with his hands, or her hands, those productive areas, those door seams or drawer seams or whatever the case may be to make sure the dog is actively sniffing in those areas and having the best opportunity to find that explosive odor in this case.
	Quote 9. If I’m looking for large hides, like you’re looking for 50-pound explosives in the trunk of a vehicle, dogs can be moving pretty quickly, and they can be sort of air searching and questing. If you’re looking for explosives that are hidden in laptops, the dog can’t be running real fast. He has to be deep down, he has to be moving at a slower pace, he has to be at a high rate of inhalation. I mean a very detailed search for that target, and I can tell that the dog is fully investigating each target of opportunity. If he runs by a suitcase, and this depends on the size of the target zone, if you’re trying to clear packages in an airport and they’re running on a conveyor belt and the dog is walking along just sniffing bags as they go by, he’s not going to find something that’s really hidden well, unless he just lucks into it. Now, big stinky hide that’s been permeating through the containers, he can catch that going by. So, the search that I want to see in the dog needs to be relative to the target that we’re seeking.
Interaction with handler	Quote 10. They can be high drive and fast, but I like to see a dog that will take direction easily and work away from the handler very well, but still doesn’t rely on the handler. So, either on or off lead, it doesn’t change the way they work, you shouldn’t see a change. They shouldn’t be relying on the handler at all is one of the things that I look for. They shouldn’t check back in too much with the handler. So, if I’m watching a dog work, I don’t particularly like when they keep checking back in with the handler, or back to people looking for those cues.
Handler requirements	Quote 11. It’s incumbent upon the individual handlers to ensure that they know their individual dogs. Not only in terms of how they respond necessarily to odor, but also the effectiveness of their dogs’ searching abilities. In other words, are there other factors that are leading these dogs to either perform well or perform poorly. So, are they tired? Have they been broken? Have they been fed? Have they been exercised? All those things go into it. And if the dog is lackadaisical and not showing any sort of interest at all in the searching activity, is there something medically wrong with the dog? That sort of thing. So, all those factors build into the dog’s ability to perform the searching task... The idea being the handlers, especially in these repetitive tasks, the handlers know what the clues are that the dog is interested in the search.

**Table 3:** Physical environment interview themes and exemplar quotations.

Interview Themes	Exemplar Quotations
Required to search nearly everywhere	Quote 12. It’s any type of building to include warehouses, barracks. They’ve got to be able to search vehicles. They’ve got to be able to search roadways, open areas. Really, the only areas that we don’t want them to get used to search in are places that would be hazardous to the dog, i.e., some sort of chemical environment or we’re not going to run a dog through some toxic sludge heap, something along those lines. If you got a ton of raw sewage laying around. Something that could make the dog sick potentially or do damage to it short and long term, that’s what we stay out of, but yeah, just something that’s unsafe to the dog is really the only limit that we have on that.
Specific locations	Quote 13. Basically, anything and everything is in play for that dog to deploy in. So, it’s just like basically any type of environment, that dog should be able to function in. I live in the mountains of (U.S. state). I’m looking at the side of a mountain right now. How many, actually outside of probably the military in Afghanistan back in the day in the mountainous regions, can work a steep grade? And how many dogs that can’t work a large grassy area without taking a piss on something? Because they have to be able to identify odor can come from any environment, not just particular ones like cars. Like they strictly do cars. Some of these private companies, all they do is cars. And then I will hear them say, “He just does cars; he can’t do rooms.” Why not? See what I’m saying? It is broad.

**Table 4:** Climate environment interview themes and exemplar quotations.

Interview Themes	Exemplar Quotations
Required to search in nearly every climate	Quote 14. I cannot picture any environments where we are not already asking dogs to work. We have dogs working outside in the winter here in various parts of the United States where it is cold. Then we’ve got them working in the swamps in Florida or in Miami, I guess, not the physical swamps but the high humidity, high temperature in Miami to the dry heat out West. I think we are already deploying them in all weather conditions from one extreme to the next.
Role of handler and leadership	Quote 15. Just because the weather is ugly, it doesn’t mean that you’re not going to have to carry on with that search. I think you need to take into consideration both as a handler and as a program manager, whoever has control of that operational environment, to look at different factors and plan for that humidity and heat.

**Table 5:** Operational environment interview themes and exemplar quotations.

Interview Themes	Exemplar Quotations
Required to settle and refocus	Quote 16. Some of us can work well with stress, some of us might be overwhelmed with whatever it is we’re feeling anxious about. It’s going to have some effect. Whether it has enough effect so the dog can’t overcome it, that’s what we want to determine. When we’re looking at dogs in our selection process, we’re putting that stress on them. We’re putting those surprises. I don’t mind the dog basically startling, but what I’m doing when I’m testing it, I’m intentionally startling the dog, but then I’m trying to see how long it takes this dog to appear to overcome it.
Manage tactical demands	Quote 17. When deployed overseas, you actually worry about somebody trying to blow you up any second. Not only are you having to work the dog, but you’re having to watch other vehicles in the area. You should have overwatch, and that’s the one nice thing about working in the military type environment, you generally have overwatch, whereas in law enforcement a lot of times you don’t. You have to worry about the people and what’s going on around you. Personal threats to the handler and the dog. Who’s the good guy, who’s the bad guy that I may have trouble with here in just a second?
Appropriate acclimation	Quote 18. If you have a really good training program, a really good setup, gunfire doesn’t bother him, loud explosives don’t bother him, people don’t bother him. They stay focused. You’ve got to have that experience within your training environment, so it becomes normal. It doesn’t shock the dog. The dog just ignores everything.
Criminal apprehension or assault training or utilization	Quote 19. Just getting your dog out of your car and going to the building, the majority of these dual purpose dogs think they’re looking for the man. Just because that’s the majority of their deployments. They’re going to alarm calls, they’re going to home invasions, burglary calls, or whatever. So, their natural instincts, until the handler can get it focused on detection, is looking for the human element, not the explosive element.
	Quote 20. If you do a call out at a house or a hit, the dog’s in bite mode which is very stimulating, and he’s looking to bite somebody. You have to say, “Let’s check these doors, let’s check these gates before we move in here, let’s clear this area so we are safe to move up.” Then you go in, and you arrest everybody, and you call them out or whatever you do, and then you say, “Okay, now we have to search for explosives again.” That switch is very difficult for the dog, to go from bite drive to explosive detection.

**Table 6:** Explosive odor environment interview themes and exemplar quotations.

Interview Themes	Exemplar Quotations
Discrimination and generalization	Quote 21. The dog’s got to be discriminate that he’s only going to alert on certain targets, but then be broad enough that he’s only going to alert on the specific precursor odor that you train on. It comes down to differences in the targets that you’re training for and the ones you’re finding. So, the dog’s ability has to be broad enough that he’s going to hit on a wide variety of ammonium nitrates, for instance, but discriminate enough that he’s not going to hit on the stuff that you’re not interested in.
Odor locations	Quote 22. You had to train these dogs that they could detect the odor in the ground. These dogs initially had problems with detection of the odor in the ground. And sometimes they would have problems with high odors, like if it was up in a tree. But, once you started training them and you started acclimating them and got them to understand, “Hey, there might be a very high odor, and I may not be able to reach it, but I’m going to get to a certain point and I’m going to respond.” With buried odor they would get extremely confused and then they would finally get it and they’d say, “Oh, okay, I got it. The odor’s in the ground and this is where it’s primarily coming from.”

## Data Availability

The raw data generated and analyzed during this study are not publicly available due to the sensitive nature of the data and ethics restrictions on data sharing. Respondents did not consent to have their data publicly shared.

## Supplementary Materials

The following are available online at https://www.mdpi.com/article/10.3390/ani11071976/s1, Interview Guide.
