# Supplementary material for: Expert Perspectives on the Performance of Explosive Detection Canines: Operational Requirements
Source: Animals (Basel). 2021 Jul 1;11(7):1976. doi: 10.3390/ani11071976 (PMC8300389; doi:10.3390/ani11071976)
Supplement: Supplementary file 1 [file animals-11-01976-s001.zip › animals-1244756-supplementary.pdf]

Operational Requirements of Explosive Detection Canines
Qualitative Interview

NAME, thank you for agreeing to do this interview. I am using a standard interview format, so let me get started.

Introduction

I am working to understand what an explosive detection canine (EDC) must do to find explosives when deployed. I am using these interviews to gain a general understanding of explosive detection requirements. The next steps are to:

- Develop a survey to learn the specific requirements from those who are currently handling EDCs.
- Analyze these results and describe the operational requirements of EDCs.
- Develop a standardized detection model which will help us better understand how an EDC does its job and how physical, mental, and environmental factors affect its performance.

Format

I will start this interview by asking you a few questions about your experience with EDCs. Then, I will ask you questions related to specific areas of explosive detection. These areas are the performance of the EDC, the physical environment, climate, the operational environment, and odor characteristics. We will only focus on your experience with explosive detection done in an operational or deployed environment rather than detection done in training.

Training

I am well aware of the role training amount, training style, and experience play in EDC performance. For the purpose of this interview, I will ignore those internal factors and focus on the external operational factors that affect every EDC.

Caveats

I am recording this interview and will later transcribe the audio to text. Please do not discuss any classified or sensitive information during this interview. None of your personally identifying information (name, specific employment information, etc.) will be associated with this interview. This interview format has been deemed exempt by the University of Pennsylvania Institutional Review Board. I will endeavour to not use your name in this interview, and if I do, it will be edited out. When answering the questions, please speak clearly. I expect this interview to take approximately 30-45 minutes. I will begin the recording now. Do I have your consent to record this interview?

Experience

I will now ask you about your experience with EDCs. I will first ask you about your experience as an EDC handler.

- Have you directly handled an operational EDC?
- If so, which category or categories best describes your experience - military, law enforcement, non-military federal agency, or private?
- If so, how many years of handling an operational EDC have you had (in each category)?
- If so, how recently did you directly handle an operational EDC?

I will now ask you about your experience as an EDC trainer.

- Have you worked as an EDC trainer?
- If so, which category or categories best describes your experience - military, law enforcement, non-military federal agency, or private?
- If so, how many years of EDC trainer experience do you have (in each category)?
- If so, how recently did you train EDCs?

I will now ask you about your experience in EDC program leadership.

- Have you worked in EDC program leadership? Examples of this include kennel master, supervisor, and program manager.
- If so, which category or categories best describes your experience - military, law enforcement, non-military federal agency, or private?
- If so, how many years of EDC program leadership do you have?
- If so, how recently did you work in EDC program leadership?

I will now ask you about other experiences with EDCs.

- Do you have other experience with EDCs?
- If so, how would you describe that experience?
- If so, which category or categories best describes your experience - military, law enforcement, non-military federal agency, private, or academia?

I will now ask you questions about various factors that influence explosive detection. Please answer these questions from your experience with explosive detection that is done in operational or deployed environments rather than in training.

EDC Performance

I will now ask you questions about your experience with EDC performance. I define performance as those observed or measured characteristics of the EDC that indicate future success or failure in identifying the target odor.

- In your experience, what characteristics of EDC performance indicate future success in identifying the target odor? In other words, how do you know when the EDC is performing well or is likely to find the odor?
- In your experience, what characteristics of EDC performance indicate future failure in identifying the target odor? In other words, how do you know when the EDC is not performing well or will be unlikely to find the odor?
- In your experience, what characteristics of EDC performance are the most significant?

Physical Environment

I will now ask you about your experience with the impact of the physical environment on detecting explosives. I define the physical environment as the manmade objects (such as buildings and vehicles) and the natural features (such as terrain and vegetation) the EDC must operate in.

- In your experience, what physical environments are EDCs required to work in?
- In your experience, which physical environments are EDCs most often required to work in?
- In your experience, what characteristics of the physical environment have you seen have the greatest positive or negative effect on the ability of EDCs?

Climate Environment

I will now ask you about your experience with the impact of the climate environment on detecting explosives. I define the climate environment as the weather-related environment the explosive detection canine must operate in.

- In your experience, what climates are EDCs required to work in?
- In your experience, which climates are EDCs most often required to work in?
- In your experience, what climate factors have you seen have the greatest positive or negative effect on the ability of explosive detection canines?

Operational Environment

I will now ask you about your experience with the impact of the operational environment on detecting explosives. I define the operational environment as the tactical situations, policies or precedents, equipment, and personnel requirements the EDC must operate in and around.

- In your experience, what operational environments are EDCs required to work in?
- In your experience, which operational environments are EDCs most often required to work in?
- In your experience, what aspects of the operational environment have you seen have the greatest positive or negative effect on the ability of EDCs?

Odor Characteristics

I will now ask you about your experience with the impact of odor characteristics on detecting explosives. I define odor characteristics as those factors that affect how the explosive will present a scent picture to the EDC.

- In your experience, what odor characteristics do EDCs encounter?
- In your experience, what odor characteristics do EDCs most often encounter?
- In your experience, what odor characteristics have you seen have the greatest positive or negative effect on the ability of EDCs?

Other Factors

I will now ask you about other factors affecting EDC operations that we haven't covered so far.

- In your experience, are there other factors that affect EDC operations?
- If so, how would you describe these factors?
- If so, which of these factors have you seen have the greatest positive or negative effect on the ability of EDCs?

Summary

Finally, I will now ask you to rank or prioritize the physical, climate, operational, and other factors that affect EDC operations.

- In your experience, which of the factors we discussed most affect EDC operations?
- In your experience, which of the factors we discussed least affect EDC operations?

This concludes our interview. I am now ending the audio recording. Thank you very much for your investment of your time and expertise to this project. Having participated in this interview, are there other subject matter experts you could recommend for participation?
